# BRD7 as key factor in PBAF complex assembly and CD8^+^ T cell differentiation

**DOI:** 10.1172/jci.insight.171605

**Published:** 2024-07-02

**Authors:** Feng Huang, Yingtong Lin, Yidan Qiao, Yaochang Yuan, Zhihan Zhong, Baohong Luo, Yating Wu, Jun Liu, Jingliang Chen, Wanying Zhang, Hui Zhang, Bingfeng Liu

**Affiliations:** 1 Institute of Human Virology, Key Laboratory of Tropical Disease Control of Ministry of Education, Guangdong Engineering Research Center for Antimicrobial Agent and Immunotechnology, Zhongshan School of Medicine, Sun Yat-sen University, Guangzhou, Guangdong, China.; 2 Guangzhou Laboratory, Guangzhou, China.; 3 Guangdong Provincial People’s Hospital, Guangdong Academy of Medical Sciences, Guangzhou, Guangdong, China.; 4 The First Clinical Medical College, Southern Medical University, Guangzhou, China.; 5 Infectious Diseases Center, Guangzhou Eighth People’s Hospital, Guangzhou Medical University, Guangzhou, China.

**Keywords:** Immunology, Infectious disease, Adaptive immunity, T cell development

## Abstract

Upon infection, naive CD8^+^ T cells differentiate into cytotoxic effector cells to eliminate the pathogen-infected cells. Although many mechanisms underlying this process have been demonstrated, the regulatory role of chromatin remodeling system in this process remains largely unknown. Here we show that BRD7, a component of the polybromo-associated BAF complex (PBAF), was required for naive CD8^+^ T cells to differentiate into functional short-lived effector cells (SLECs) in response to acute infections caused by influenza virus or lymphocytic choriomeningitis virus (LCMV). BRD7 deficiency in CD8^+^ T cells resulted in profound defects in effector population and functions, thereby impairing viral clearance and host recovery. Further mechanical studies indicate that the expression of BRD7 significantly turned to high from naive CD8^+^ T cells to effector cells, which bridged BRG1 and PBRM1 to the core module of PBAF complex, consequently facilitating the assembly of PBAF complex rather than BAF complex in the effector cells. The PBAF complex changed the chromatin accessibility at the loci of *Tbx21* gene and upregulated its expression, leading to the maturation of effector T cells. Our research demonstrates that BRD7 and the PBAF complex are key in CD8^+^ T cell development and present a significant target for advancing immune therapies.

## Abstract 2

BRD7 facilitates differentiation of CD8^+^ T cells into functional short-lived effector cells in response to viral infection and is required for viral clearance and host recovery.

## Introduction

CD8^+^ T cells play critical roles in protective immunity against intracellular pathogens including viruses. In response to viral infection, naive CD8^+^ T cells rapidly undergo a pronounced clonal expansion and differentiate into antigen-specific effector cells to eliminate infected cells (1, 2). During this process, effector CD8^+^ T cells acquire the ability to produce cytolytic and effector cytokines such as granzyme B (Gzmb) and IFN-γ (2–4). Within the effector populations, many effector CD8^+^ T cells are short-lived effector cells (SLECs), which undergo programmed cell death, leaving behind a small population of memory precursor effector cells (MPECs) (5). These 2 subsets are divided by 2 critical surface markers, KLRG1 and IL-7R (CD127). SLECs express high levels of KLRG1 and low levels of IL-7R and exhibit higher expression of effector molecules, while MPECs express high levels of IL-7R and low levels of KLRG1 and show a greater stem-cell like properties. The clonal expansion and effector differentiation of CD8^+^ T cells are regulated by various transcription factors. Several transcription factors, including T-bet, Blimp-1, Id2, and IRF4, are critical regulators for the differentiation of the SLEC population (5–9), while Eomes, Foxo1, Id3, and Tcf1 are required for the differentiation of the MPEC population (8, 10–13). The epigenetic and chromatin states also influence the differentiation of SLECs and MPECs (14, 15). However, more epigenetic mechanisms by which SLECs become committed to a terminal fate remain to be elucidated.

Switch/sucrose nonfermentable (SWI/SNF) chromatin-remodeling complexes contain members of ATPases that regulate DNA-protein contacts through energy generated from ATP hydrolysis. These ATP-dependent chromatin remodeling complexes are multimeric molecular assemblies involved in the regulation of chromatin architecture (16–19). Previous studies have revealed that SWI/SNF complexes in mammalian cells exist in 3 nonredundant assembly bodies: BRG1/BRM-associated factor complex (BAF), polybromo-associated BAF complex (PBAF), and noncanonical BAF complex (ncBAF) (17). SWI/SNF complexes include many core components (20). Among them, SMARCB1, SMARCC1, SMARCC2, SMARCD1, and SMARCE1 are the components of “core module,” while BRG1 (also known as SMARCA4) belong to the ATPase module of the complexes. All of these modules are shared by both BAF and PBAF complexes. Besides its important role in cancers, BRG1 is also important for T cell differentiation, since BRG1 deficiency in mice results in thymic abnormalities and a developmental block at double-negative to double-positive T cell transition (21, 22). The specific components of the BAF complex include ARID1A, ARID1B, and DPF2, while the specific components of the PBAF complex include bromodomain-containing protein 7 (BRD7), PBRM1, PHF10, and ARID2 (17, 20, 23, 24). How the ATPase module and the BAF core module are assembled together and the role played by these special components remain to be determined.

BRD7 belongs to the bromodomain family and can recognize and bind to acetylated histone H (25–27). It is ubiquitously expressed in the nucleus and only belongs to the PBAF complex (25, 28). BRD7 has been reported as a transcriptional regulator and plays a critical role in cellular growth, cell cycle progression, and tumor development (28–36). Along with its PBAF partners, BRD7 participates in β cell regulation or the resistance of tumor cells to immune cells (37, 38). Depletion of these genes in tumor cells would enhance the secretion of various chemokines, which help recruit effector T cells (38). Although the interactions among components within PBAF complex have been largely determined in a recent biochemical study (17), the role played by BRD7 in assembling the PBAF complex and how this complex is assembled to accomplish its function within the immune cells remain to be determined.

In order to investigate the function of BRD7 and its PBAF complex in antiviral immune response, we utilized mice with T cell–specific genetic deficiencies of BRD7 and infected these mice and their littermate WT controls with influenza viruses or lymphocytic choriomeningitis virus (LCMV) Armstrong (Arm) viruses, and we examined the influence of BRD7 upon T cell function. We found that the expression of BRD7 was significantly induced in effector CD8^+^ T cells. BRD7 was not required for the early activation and expansion of CD8^+^ T cells but was critical for effector differentiation of CD8^+^ T cells and pathogen clearance during acute viral infection. BRD7-deficient CD8^+^ T cells failed to initiate the effector T cell transcriptional program and showed impaired cytotoxicity and cytokine production. We therefore demonstrate that BRD7 controlled the differentiation of cytotoxic effector CD8^+^ T cells. Importantly, we identified that BRD7 played a key role in assembling the PBAF complex to perform their functions in effector CD8^+^ T cells.

## Results

### Increased expression of BRD7 in the effector CD8^+^ T cells.

**Figure 1:**
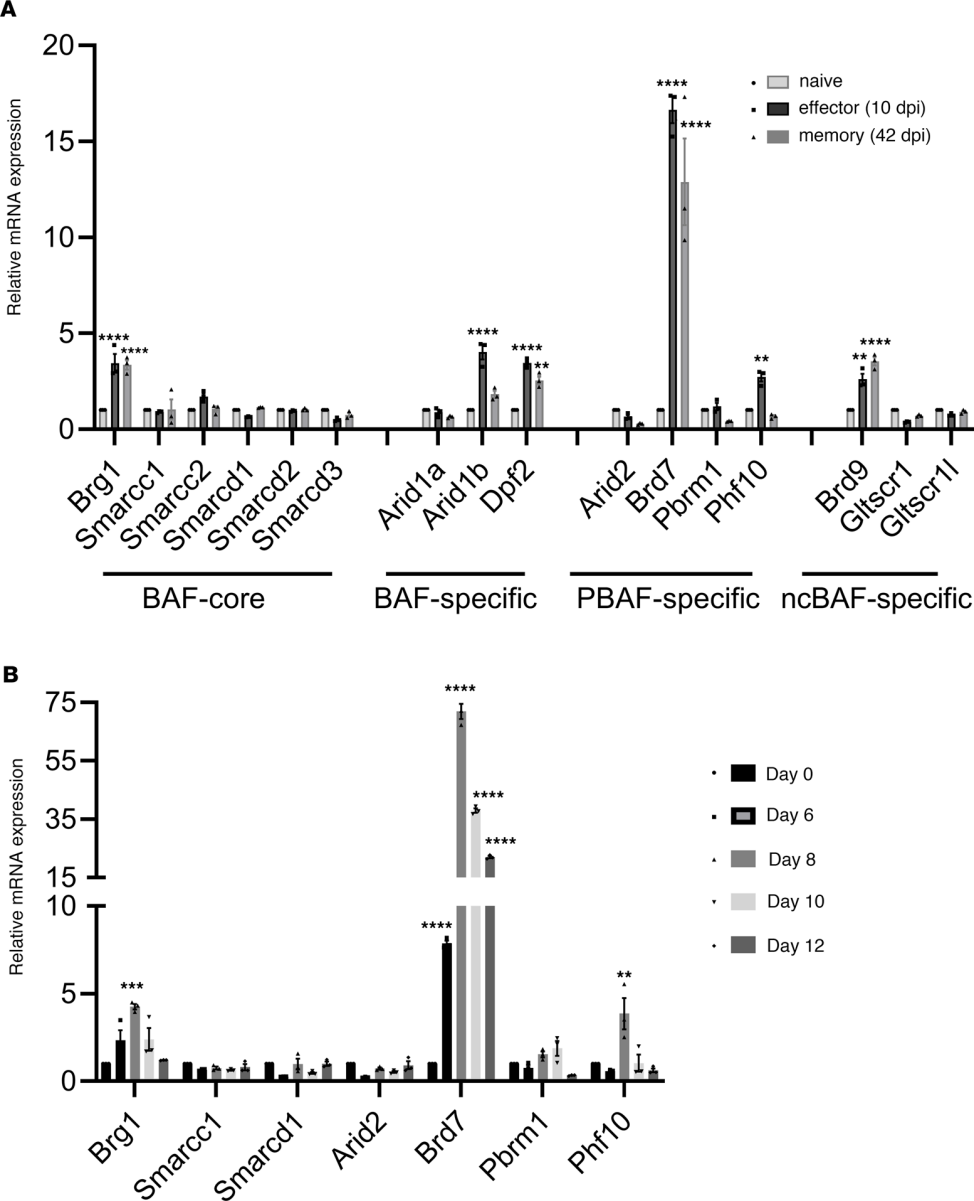
BRD7 is upregulated in effector CD8^+^ T cells. (**A**) Differential expression of relative mRNAs in naive, effector, and memory OT-I CD8^+^ T cells was analyzed. Naive OT-I cells purified from spleen of OT-I mice were transferred into recipient CD45.1 mice and then infected with PR8-OVA at day 1 after transfer. Effector (10 days [d] p.i.) and memory (~42 d p.i.) OT-I cells were purified with FACS for mRNA quantification relative to that of naive OT-I cells. (**B**) Time course of mRNA expression of several PBAF components in OT-I T cells purified from spleen of OT-I mice, which were transferred into recipient CD45.1 mice and then infected with PR8-OVA at day 1 after transfer. **P < 0.01, ***P < 0.001, and ****P < 0.0001 (2-way ANOVA). Mean ± SEM of 3 mice per group. Data are representative of 2 independent experiments.

The SWI/SNF complexes are involved in genome-wide transcriptional regulation (16, 17). However, their roles in antiviral immune response remain unknown. To elucidate the roles of SWI/SNF chromatin remodeling complexes in regulating CD8^+^ T cell responses, we analyzed the expression of components of BAF, PBAF, and ncBAF complexes after influenza virus infection. Among these complex components, we found that the expression of BRD7 was the highest in effector CD8^+^ T cells at day 10 postinfection (p.i.), and the expression maintained high in memory CD8^+^ T cells (at ~42 days p.i.) from OT-I TCR-transgenic mice when compared with their mRNA expression in naive CD8^+^ T cells (Figure 1A). In addition, when we analyzed the kinetics of mRNA expression of several PBAF components during in vivo responses to infection, we found that *Brd7* mRNA expression was upregulated at day 6 p.i. and reached the maximum at day 8 (Figure 1B), suggesting that the expression of BRD7 is concurrent with the development of effector CD8^+^ T cells. Of note, the CD8^+^ T cells of these mice recognize a peptide fragment of chicken ovalbumin (OVAp) bound to H-2K^b^ when infected with PR8-OVA virus, a recombinant strain of A/PR/8/34 (H1N1) virus expressing OVA. These data therefore indicate that BRD7 in CD8^+^ T cells may play a critical role in regulating immune response.

In order to examine the role of BRD7 in CD8^+^ T cell response, we generated mice that conditionally deleted *Brd7* in T cell–specific conditional BRD7-deleted mice (*Brd7*^ΔT^) by crossing mice homozygous for *lox*P-flanked alleles of *Brd7* (*Brd7^fl/fl^*) to mice expressing a transgenic encoding Cre recombinase from T cell–specific *Cd4* promoter (*Cd4-Cre*) (Supplemental Figure 1; supplemental material available online with this article; https://doi.org/10.1172/jci.insight.171605DS1); this resulted in lacking expression of BRD7 in mature CD8^+^ T cells (Supplemental Figure 2A). The thymic T cell development and T cell circulation were not affected in *Brd7*^ΔT^ mice since similar percentages and absolute numbers of CD4^+^ and CD8^+^ T cells were found in thymus, mediastinal lymph nodes (mLN), and spleen from both BRD7 WT mice (*Brd7^fl/fl^*) and BRD7-deleted littermate pairs (*Brd7*^ΔT^) (Supplemental Figure 2, B and C). To explore the potential for any off-target effects on related Brd family proteins, we carried out Western blotting to scrutinize their expression. The findings revealed that, with the exception of *Brd7*, the expression of *Brd2*, *Brd4*, *Brg1*, and *Trim-28* remained unaltered in the BRD7-deficient mice (Supplemental Figure 3). Thus, this T cell–specific *Brd7*-deleted mice allowed us to specifically analyze the role of BRD7 in CD8^+^ T cell responses during infection.

### BRD7 deficiency impairs the differentiation of effector CD8^+^ T cells.

**Figure 2:**
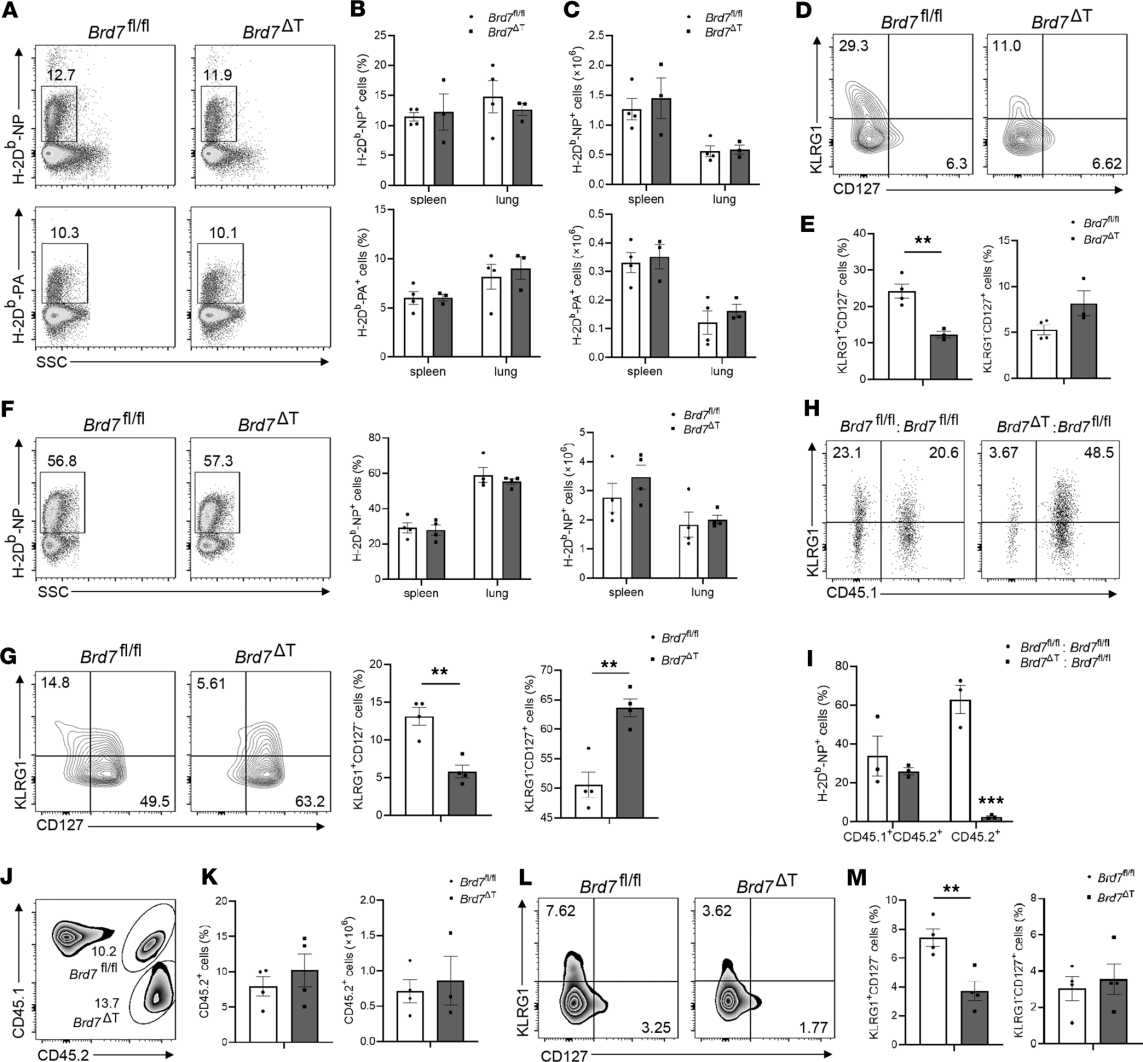
BRD7 controls SLEC differentiation. (**A**) Flow cytometry of influenza H-2D^b^-NP^+^CD8^+^ and H-2D^b^-PA^+^CD8^+^ T cells from spleen and lungs of BRD7 WT (*Brd7^fl/fl^*) (*n* = 4) and BRD7-deficient (*Brd7*^ΔT^) (*n* = 3) mice. Mice were infected with influenza HKx31 virus, and NP366 and PA244 tetramer were stained in lung or spleen CD8^+^ T cells at 10 d p.i. (**B** and **C**) Frequency and cell number of H-2D^b^-NP^+^ or H-2D^b^-PA^+^ cells among CD8^+^ T cells in **A**. (**D** and **E**) Flow cytometry of KLRG1 and CD127 on H-2D^b^-NP^+^CD8^+^ T cells in the lungs obtained from *Brd7^fl/fl^* (*n* = 4) or *Brd7*^ΔT^ (*n* = 3) mice infected with HKx31 at 10 d p.i. (**F**) Flow cytometry of H-2D^b^-NP^+^CD8^+^ T cells from spleen and lungs of PR8-primed *Brd7^fl/fl^* (*n* = 4) and *Brd7*^ΔT^ (*n* = 4) mice rechallenged with HKx31 at 6 weeks after PR8 infection. (**G**) Flow cytometry of KLRG1 and CD127 on H-2D^b^-NP^+^CD8^+^ T cells in the spleen obtained of PR8-primed *Brd7^fl/fl^* (*n* = 4) and *Brd7*^ΔT^ (*n* = 4) mice rechallenged with HKx31 at 6 weeks after PR8 infection. (**H** and **I**) Flow cytometry of KLRG1 and CD45.1 on NP^+^CD8^+^ T cells from chimeras. Mixed bone marrow chimeras were generated by reconstitution of lethally irradiated CD45.1 mice with bone marrow from *Brd7^fl/fl^* (CD45.2^+^) plus *Brd7^fl/fl^* (CD45.1^+^CD45.2^+^) (*n* = 3), or *Brd7*^ΔT^ (CD45.2^+^) plus *Brd7^fl/fl^* (CD45.1^+^CD45.2^+^) (*n* = 3) at a ratio of 1:1. Chimeras were infected with HKx31 at 6 weeks after reconstitution and were sacrificed at 10 d p.i. for analysis. (**J**–**M**) Flow cytometry of OT-I CD8^+^ T cells (**J** and **K**) or KLRG1^+^CD127^–^ SLECs and KLRG1^–^CD127^+^ MPECs (**L** and **M**) in CD45.1 host mice (*n* = 4) 9 days after transfer of *Brd7^fl/fl^* (CD45.1^+^CD45.2^+^) plus *Brd7*^ΔT^ (CD45.2^+^) OT-I CD8^+^ T cells at a ratio of 1:1 and infection with PR8-OVA virus 1 day after transfer. Data are shown as mean ± SEM. **P < 0.01 (2-tailed Student’s *t* test). Data are representative of 3 independent experiments.

To examine the response of CD8^+^ T cell subsets of BRD7-sufficient and BRD7-deficient mice during virus infection, we infected *Brd7^fl/fl^* mice and *Brd7*^ΔT^ mice with the influenza HKx31 strain and analyzed the CD8^+^ T cells in the spleen and lungs at the peak of the response (day 10 p.i.). We analyzed influenza antigen–specific CD8^+^ T cell responses by staining the H-2D^b^-NP (NP_366–374_) and H-2D^b^-PA (PA_224–233_) tetramers. There were no obvious differences in the percentages and numbers of the influenza-specific H-2D^b^-NP^+^CD8^+^ and H-2D^b^-PA^+^CD8^+^ T cells in the spleen and lungs from *Brd7^fl/fl^* mice and *Brd7*^ΔT^ mice (Figure 2, A–C). However, the frequency of antigen-specific KLRG1^+^IL-7R^–^ SLECs was significantly lower in BRD7-deficient CD8^+^ T cells than in BRD7 WT CD8^+^ T cells (Figure 2, D and E). This finding was not due to tissue distribution, since similar results were found in both spleen (Supplemental Figure 4A) and the lung (Figure 2, D and E). Antigen-specific BRD7-deficient CD8^+^ T cells also showed increased expression of CD62L and CD27 (Supplemental Figure 4B), a defective SLEC differentiation phenotype (7, 39). This phenotype was not due to abnormal activation of CD8^+^ T cells, since the expression of CD69 and CD44 were not altered in BRD7-deficient CD8^+^ T cells (Supplemental Figure 4B). Likewise, to investigate the role of BRD7 in a secondary effector CD8^+^ T cell response, we used a 2-infection model in our study. In the primary infection, mice were infected i.n. with the influenza PR8 virus (H1N1 virus). In the secondary infection, mice were rechallenged with the HKx31 virus i.n. 6 weeks after the first infection. We found that the frequency of NP^+^CD8^+^ T cells in the lung and spleen was similar between *Brd7^fl/fl^* mice and *Brd7*^ΔT^ mice (Figure 2F), while a reduction of antigen-specific KLRG1^+^IL-7R^–^ SLECs was found in the *Brd7*^ΔT^ mice after infection with HKx31 viruses of previously PR8-primed mice (Figure 2G). These results indicate a defect in the primary and secondary effector differentiation of CD8^+^ T cells without BRD7.

To explore whether the defective differentiation of SLECs in BRD7-deficient mice depended on the direct role of BRD7 in CD8^+^ T cells, we generated chimeras by reconstituting lethally irradiated CD45.1 mice with a various 1:1 mixture of bone marrow cells of *Brd7^fl/fl^* mice and *Brd7*^ΔT^ mice, followed by infection with HKx31 at 6 week after reconstitution. The frequency of antigen-specific effector CD8^+^ T cells was analyzed in the spleen of chimeras. We found that the BRD7-deficient CD8^+^ T cells produced much lower antigen-specific KLRG1^+^ effector cells than BRD7 WT CD8^+^ T cells, even in the presence of WT CD8^+^ T cells (Figure 2, H and I), and this suggested that BRD7 influenced the differentiation of SLECs depending on the intrinsic role of BRD7 in CD8^+^ T cells. Furthermore, to rule out the possibility that the deletion of BRD7 in CD4^+^ T cells may affect the phenotype, we crossed BRD7-deficient mice with mice of the OT-I TCR-transgenic strain to generate OT-I BRD7-deficient mice. The mixture of OT-I BRD7-deficient naive CD8^+^ T cells (CD45.2^+^) and OT-I BRD7 WT naive CD8^+^ T cells (CD45.1^+^CD45.2^+^) at a ratio of 1:1 was adoptively transferred into CD45.1 mice, followed by infection with PR8-OVA viruses after 24 hours. We found that OT-I BRD7-deficient CD8^+^ T cells failed to generate as many SLECs as OT-I BRD7 WT CD8^+^ T cells did (Figure 2, J–M). Altogether, these results show that BRD7 regulated the differentiation in a CD8^+^ T cell–intrinsic manner.

### BRD7 is required for the effector function of CD8^+^ T cells.

**Figure 3:**
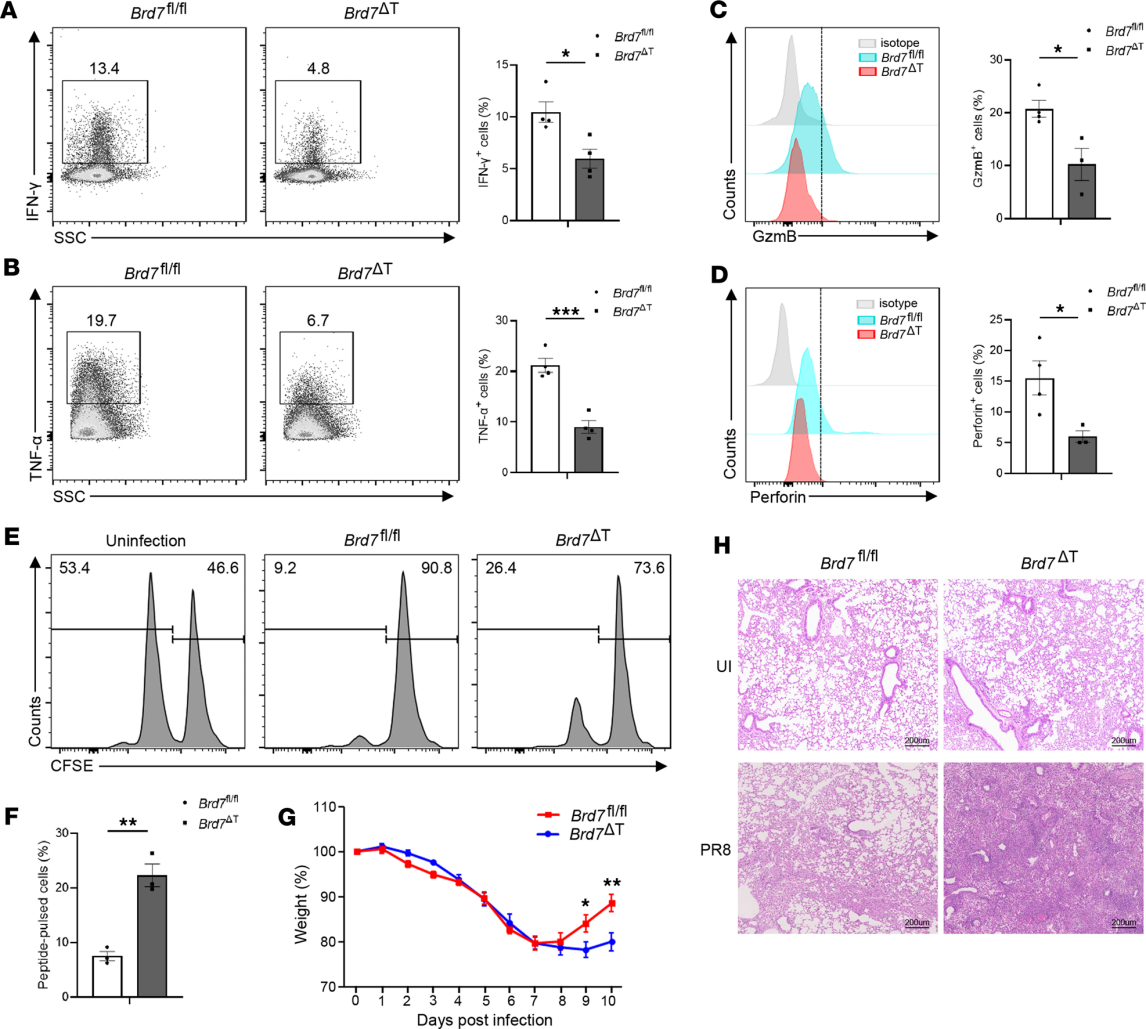
BRD7 is required for effector function. (**A** and **B**) Intracellular cytokine staining of IFN-γ (**A**) or TNF-α (**B**) produced by splenic CD8^+^ T cells from *Brd7^fl/fl^* (*n* = 4) and *Brd7*^ΔT^ (*n* = 4) mice infected with HKx31 virus stimulated with NP peptide at 10 d p.i. Number beside outlined areas indicates percent of IFN-γ^+^CD8^+^ (**A**) or TNF-α^+^CD8^+^ (**B**) T cells. Frequency of IFN-γ^+^ (**A**) or TNF-α^+^ (**B**) cells among CD8^+^ T cells. (**C** and **D**) Flow cytometry of granzyme B (GzmB) (**C**) or perforin (Prf1) (**D**) produced by splenic NP^+^CD8 ^+^ T cells from *Brd7^fl/fl^* (*n* = 4) and *Brd7*^ΔT^ (*n* = 3) mice infected with HKx31 virus at 10 d p.i. Numbers beside outlined areas indicate mean fluorescence intensity (MFI) of GzmB (**C**) or Prf1 (**D**) among NP^+^CD8^+^ T cells. MFI of GzmB (**C**) or Prf1 (**D**) among NP^+^CD8^+^ T cells. (**E**) In vivo cytolysis assay. Noninfected WT host mice or infected BRD7 WT (*n* = 3) or BRD7-deficient (*n* = 3) mice received equal numbers of low CFSE–labeled B6 splenocytes loaded with NP peptide plus high CFSE–labeled B6 splenocytes without NP peptide stimulation at 10 d p.i. Cytotoxic T lymphocyte activity was assessed 4 hours after transfer. Numbers above bracketed lines represent percentages of cells per CFSE peak. (**F**) Frequency of peptide-pulsed cells in **E** was shown. (**G**) Body weight of *Brd7^fl/fl^* (*n* = 8) and *Brd7*^ΔT^ (*n* = 7) mice infected with 0.5 LD_50_ of A/PR/8/34 (H1N1) virus at various times p.i. Body weight at day 0 was set as 100%. (**H**) Histological examination of lung from *Brd7^fl/fl^* or *Brd7*^ΔT^ mice noninfected with PR8 virus (top panel) or infected at day 8 p.i. (bottom panel). Lung from *Brd7*^ΔT^ mice infected with PR8 virus showed severe tissue damage and lymphocytic infiltration. Data are shown as mean ± SEM. *P < 0.05, **P < 0.01, and ***P < 0.001 (2-tailed Student’s *t* test). Data are representative of 3 independent experiments.

After exposure to antigen, activated CD8^+^ T cells undergo an effector differentiation process and gain the functional ability to produce cytotoxic and effector cytokines to eliminate intracellular pathogens. To examine whether BRD7 deficiency affects the effector functions, we measured cytokine production from CD8^+^ T cells after NP-peptide stimulation by intracellular staining and flow cytometric analysis. The proportion of BRD7-deficient CD8^+^ T cells producing IFN-γ (Figure 3A) and TNF-α (Figure 3B) was significantly decreased. Likewise, the production of the cytolytic effector molecule GzmB (Figure 3C) and perforin (Figure 3D) was hardly detected in antigen-specific BRD7-deficient CD8^+^ T cells. Low production of these cytolytic effector cytokines will result in a defect in the ability to kill targets. Indeed, BRD7-deficient CD8^+^ T cells showed a diminished cytolytic effector function in an in vivo cytolysis assay (Figure 3, E and F). Of note, in the in vivo cytolysis experiment, we stained splenocytes stimulated with NP peptide by low-dose carboxyfluorescein diacetate succinimidyl ester (CFSE^lo^ cells) and splenocytes without NP peptide stimulation by high-dose CFSE (CFSE^hi^ cells). Then, we mixed CFSE^lo^ cells and CFSE^hi^ cells at a ratio of 1:1 and injected the mixture into WT mice that were not infected with HKx31, WT mice infected with HKx31, or BRD7-deficient mice infected with HKx31 separately. Almost complete clearance of NP-pulsed cells was observed in WT mice infected with HKx31, whereas the killing of these cells was less effective in BRD7-deficient mice infected with HKx31. Similarly, *Brd7*^ΔT^ mice showed a higher degree of weight loss (Figure 3G) and more severe pathology in the lung than *Brd7^fl/fl^* mice did after infection with the aggressive influenza PR8 viruses (Figure 3H). Taken together, these data suggest that BRD7 was required for the production of functional CD8^+^ effector T cells and the clearance of viruses.

**Figure 4:**
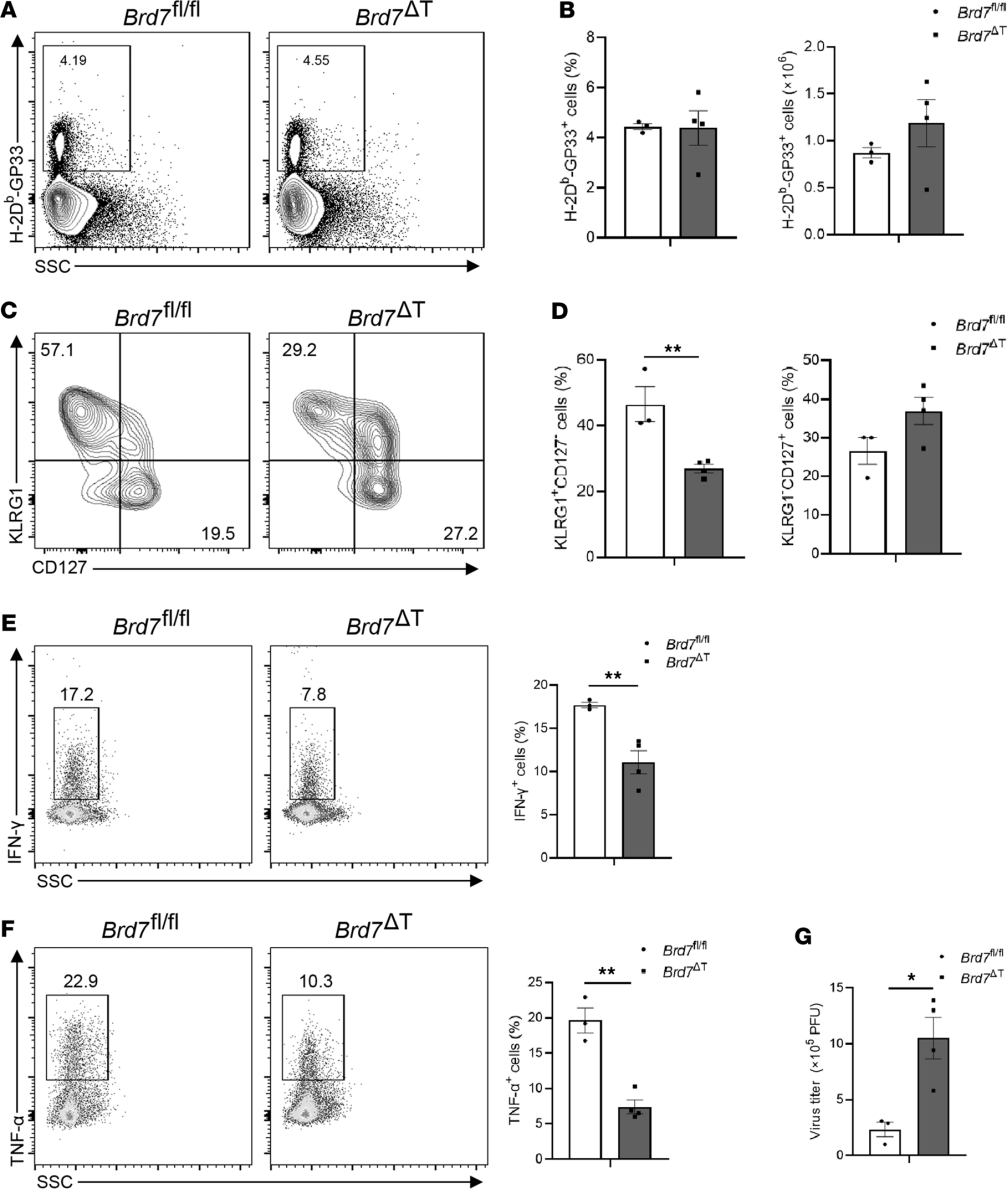
BRD7 controls SLEC differentiation during LCMV infection. (**A**) Flow cytometry of Arm gp33^+^CD8^+^ T cells from spleen of *Brd7^fl/fl^* (*n* = 3) and *Brd7*^ΔT^ (*n* = 4) mice. *Brd7^fl/fl^* and *Brd7*^ΔT^ mice were infected with LCMV-Arm virus, and antigen-specific gp33 tetramer was stained in spleen CD8^+^ T cells at day 8 p.i. Numbers beside outlined areas indicate percent of gp33^+^CD8^+^ T cells. (**B**) Frequency (left) and cell number (right) of gp33-specific cells among CD8^+^ T cells in **A**. (**C**) Flow cytometry of KLRG1 and CD127 on gp33^+^CD8^+^ T cells in the spleen obtained from *Brd7^fl/fl^* (*n* = 3) or *Brd7*^ΔT^ (*n* = 4) mice infected with Arm at 8 d p.i. Numbers in quadrants indicate percent of KLRG1^+^CD127^–^ SLECs (top left) or KLRG1^–^CD127^+^ MPECs (bottom right). (**D**) Frequency of KLRG1^+^CD127^–^ SLECs or KLRG1^–^CD127^+^ MPECs among gp33^+^CD8^+^ T cells in **C**. (**E** and **F**) Intracellular cytokine staining of IFN-γ (**E**) or TNF-α (**F**) produced by splenic CD8^+^ T cells from *Brd7^fl/fl^* (*n* = 3) and *Brd7*^ΔT^ (*n* = 4) mice infected with Arm virus stimulated with gp33 peptide at day 8 p.i. Number beside outlined areas indicates percentage of IFN-γ^+^CD8^+^ (**E**) or TNF-α^+^CD8^+^ (**F**) T cells. Frequency of IFN-γ^+^ (**E**) or TNF-α^+^ (**F**) cells among CD8^+^ T cells in left. (**G**) LCMV Arm virus titers in the spleen of *Brd7^fl/fl^* (*n* = 3) or *Brd7*^ΔT^ (*n* = 4) mice infected with LCMV Arm strain were determined at day 8 p.i. Data are shown as mean ± SEM. *P < 0.05, **P < 0.01 (2-tailed Student’s *t* test). Data are representative of 3 independent experiments.

CD8^+^ T cells will proliferate and differentiate into effector cells to eradicate pathogen during virus infection. This process should be independent of virus strain. To further demonstrate the role of BRD7 in CD8^+^ T cell responses to other viral infection, we further utilized the LCMV infection model in our study, which initiates an acute infection. We infected *Brd7^fl/fl^* mice or *Brd7*^ΔT^ mice with the Arm strain of LCMV, and we analyzed genotypes of mice at the peak of the response (day 8 p.i.). Similar percentages and numbers of the LCMV gp33-H-2D^b^–specific CD8^+^ T cells were detected in *Brd7^fl/fl^* and *Brd7*^ΔT^ mice (Figure 4, A and B), whereas the proportion of antigen-specific KLRG1^+^IL-7R^–^ SLECs was fewer in *Brd7*^ΔT^ mice when compared with littermate controls (Figure 4, C and D). Also, a defect in the production of effector cytokines was detected in CD8^+^ T cells from *Brd7*^ΔT^ mice infected with LCMV (Figure 4, E and F). Moreover, a reduced clearance of LCMV virus was observed in *Brd7*^ΔT^ mice compared with littermate controls (Figure 4G). These results indicate that BRD7 mediated the SLEC development independently of virus strain or TCR specificity.

### BRD7 regulates the expression of genes critical for effector differentiation.

Cell proliferation and death control the expansion of CD8^+^ T cells. To explore the proliferation of CD8^+^ T cells in vivo after infection, we evaluated the 5-bromodeoxyuridine (BrdU) incorporation in antigen-specific cells at day 10 after HKx31 infection. We found that the BrdU incorporation of NP^+^CD8^+^ T cells was similar in BRD7-deficient and BRD7 WT mice (Supplemental Figure 5A), indicating that BRD7 deficiency did not alter the proliferation of antigen-specific CD8^+^ T cells. In addition, when we quantify the apoptotic CD8^+^ T cells from infected mice using annexin V and propidium iodide staining, we did not find notable differences of the apoptotic antigen-specific CD8^+^ T cells between BRD7-deficient and WT mice (Supplemental Figure 5B). These data suggest that BRD7 did not mediate cell proliferation or apoptosis of CD8^+^ T cells, which is consistent with the above data that BRD7 deficiency did not change the proportion of antigen-specific CD8^+^ T cells.

**Figure 5:**
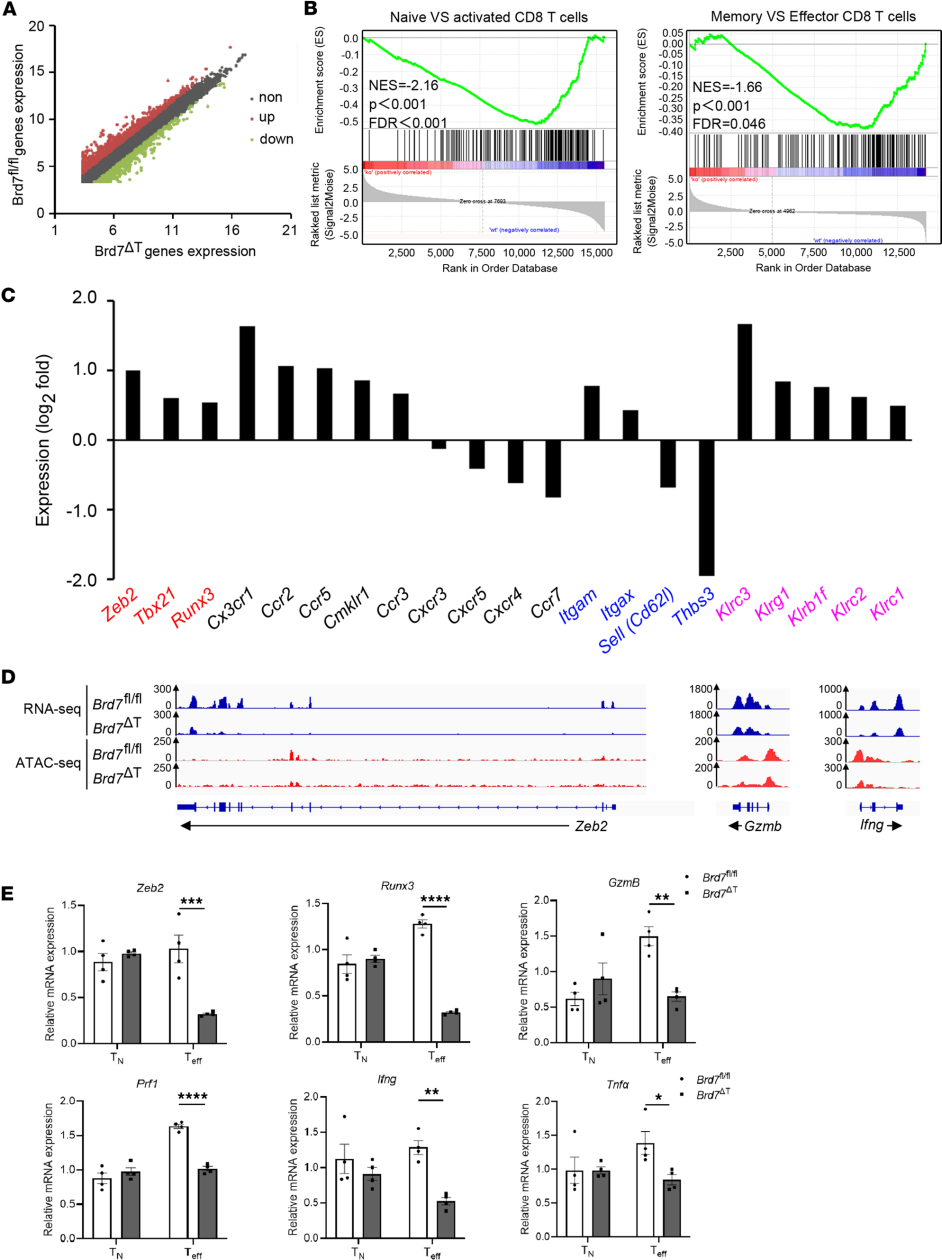
BRD7 orchestrates the expression of genes critical for SLEC differentiation. (**A**) Scatter plot analysis of differentially expressed genes of NP^+^CD8^+^ T cells from *Brd7^fl/fl^* and *Brd7*^ΔT^ mice infected with HKx31 at 10 d p.i. FDR ≤ 0.05 and log_2_ fold change ≥ 2 were used as the threshold to evaluate the significance of differences in gene expression. (**B**) Gene set enrichment analysis (GSEA) in **A** among transcriptional differences between naive and activated CD8^+^ T cells (left) or memory and effector cells (right). NES, normalized enrichment score. (**C**) Differentially expressed genes of transcription factor (red), chemokine receptors (black), adhesion molecules (blue), and killer cell lectin-like receptors (pink) between WT and BRD7-deficient cells, represented as the log_2_ fold change. The *y* axis displays the log_2_ fold change of each DEG, and the *x* axis lists the gene name. (**D**) Genome browser tracks displaying RNA-Seq and ATAC-Seq data at a selected locus comparing WT and BRD7-deficient cells. Tag density from different groups was calculated by normalizing to the total mapped reads. (**E**) qPCR analysis of mRNA in BRD7-deficient naive CD8^+^ T cells (T_N_) or NP^+^CD8^+^ T cells (T_eff_) (*n* = 4) presented relative to expression in BRD7 WT cells (*n* = 4). Data are shown as mean ± SEM. *P < 0.05, **P < 0.01, ***P < 0.001, and ****P < 0.0001 (2-tailed Student’s *t* test). Data are representative of 2 independent experiments.

To better elucidate the underlying molecular mechanism by which BRD7 controls the differentiation of SLECs, we analyzed the global gene expression profiles of BRD7 WT and BRD7-deficient antigen-specific CD8^+^ T cells using RNA-Seq. We sorted NP^+^CD8^+^ T cells from *Brd7^fl/fl^* mice and *Brd7*^ΔT^ mice infected with HKx31 by flow cytometry at day 10 p.i. BRD7 deficiency led to upregulation of 856 genes and downregulation of 647 genes compared with their WT counterparts (Figure 5A). The differential expressed genes were highly enriched among transcripts induced in effector CD8^+^ T cells and activated CD8^+^ T cells (Figure 5B). We also observed a notably different expression in SLEC signature genes, including *Zeb2*, *Tbx21*, *Sell* (*Cd62l*), and *Klrg1* (Figure 5C). These genes were further categorized as transcription factors, chemokine receptors, adhesion molecules, and killer cell lectin-like receptors (Figure 5C), indicating that BRD7 exerted broad regulatory effects upon SLEC signature genes.

As an essential component of the PBAF complex, BRD7 deficiency may result in the change of chromatin structure. To test this possibility with a higher-resolution method, we performed assay of transposase assessable chromatin-sequencing (ATAC-Seq), which detects the insert of Tn5 transposase in open chromatin regions (40). ATAC-Seq analysis of NP^+^CD8^+^ T cells showed a less accessible chromatin configuration at SLEC signature genes, including *Zeb2*, *Gzmb*, and *Ifng*, in cells from *Brd7*^ΔT^ mice than that from *Brd7^fl/fl^* mice (Figure 5D), and this was consistent with RNA-Seq data. The different expression of some important molecules involved in the differentiation of SLECs was further validated by quantitative PCR (qPCR) (Figure 5E). Consistent with RNA-Seq and ATAC-Seq data, all these genes were decreased in BRD7-deficient cells compared with their WT counterparts in the effector CD8^+^ T cells but not in naive CD8^+^ T cells. Together, these results reveal that BRD7 was a major regulator of genes involved in SLEC differentiation and function.

### The PBAF complex enriches at Tbx21 promoter and affects T-bet expression in effector CD8^+^ T cells.

**Figure 6:**
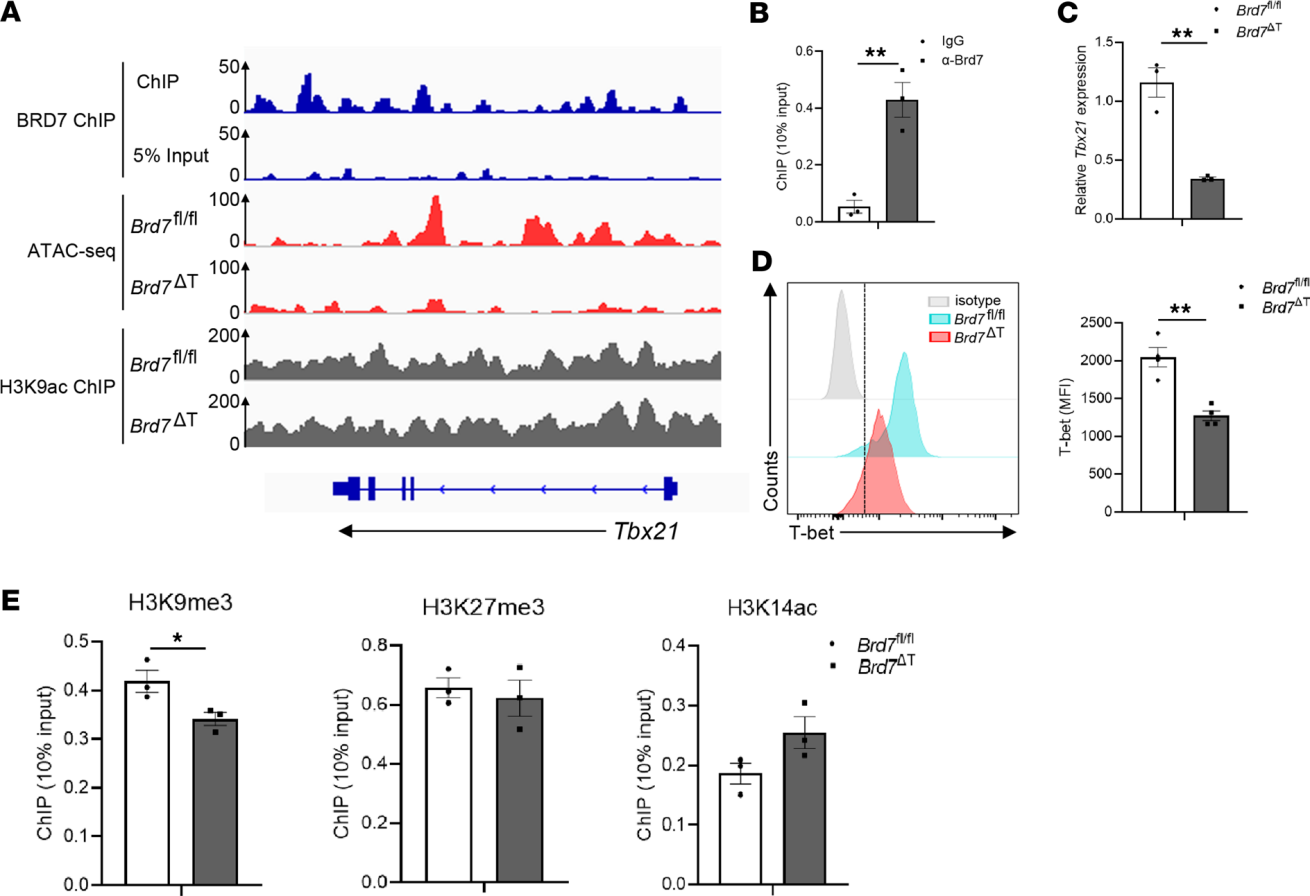
BRD7 is enriched at *Tbx21* loci and regulates T-bet expression. (**A**) Representative alignments of ChIP-, ATAC- and RNA-Seq measurements at *Tbx21* loci. ATAC- and RNA-Seq measurements from cells in Figure 5. ChIP-Seq with antibody to BRD7 was assessed with OT-I CD8^+^ T cells from OT-I mice infected with PR8-OVA at day 8 p.i. ChIP-Seq with antibody to H3K9ac was assessed with OT-I CD8^+^ T cells from *Brd7^fl/fl^* and *Brd7*^ΔT^ OT-I mice infected with PR8-OVA at day 8 p.i. (**B**) ChIP analysis (*n* = 3) shows the deposition of BRD7 at the promoter regions of *Tbx21* loci. (**C**) qPCR analysis of *T-bet* mRNA in BRD7-deficient NP^+^CD8^+^ T cells (*n* = 3) presented relative to expression in BRD7 WT cells (*n* = 3). (**D**) Flow cytometry of T-bet in BRD7 WT (*n* = 4) or BRD7-deficient (*n* = 4) NP^+^CD8^+^ T cells. Frequency of T-bet expressing NP^+^CD8^+^ T cells. (**E**) ChIP-PCR assay shows the deposition of H3K9me3, H3K27me3, and H3K14ac at the promoter regions of *Tbx21* loci. *Brd7^fl/fl^* mice (*n* = 3) and *Brd7*^ΔT^ mice (*n* = 3) were used in the experiments. Data are shown as mean ± SEM. *P < 0.05 and **P < 0.01 (2-tailed Student’s *t* test). Data are representative of 2 independent experiments.

Based on the fact that BRD7 is the specific component of the PBAF complex, a chromatin remodeling complex, we further examined the chromatin state of the BRD7-bound regions by ChIP-Seq with antibodies to BRD7 in BRD7 WT OT-I CD8^+^ T cells from OT-I mice infected with PR8-OVA at day 8 p.i. Notably, we found that BRD7 was enriched at the *Tbx21* loci (Figure 6A), and this was further validated with ChIP-PCR (Figure 6B). T-bet is a major driver for CD8^+^ T cell SLEC lineage commitment and controls the expression of a large number of molecules critical for the effector differentiation of CD8^+^ T cells, including *Zeb2*, *Runx3*, *Ifng*, *Gzma*, and *Prf1* (5, 41–44). The loss of T-bet leads to abrogated cytotoxic function and influences nearly 50% of the SLEC-specific genes. We hypothesized that BRD7 bound to the *Tbx21* loci and regulated T-bet expression. Indeed, RNA-Seq and ATAC-Seq revealed a defect in transcriptional activity of the *Tbx21* gene after BRD7 deficiency (Figure 5 and Figure 6A). A significant decrease of T-bet expression in BRD7-deficient NP^+^CD8^+^ T cells was also observed by qPCR (Figure 6C) and flow cytometry analysis (Figure 6D). Taken together, our results suggest that T-bet acts as the downstream factor for BRD7 to drive during the differentiation of effector cells.

BRD7 was previously reported to bind to H3K9ac (26). To test the possibility that BRD7 binds to H3K9ac and increases the H3K9ac deposition at the *Tbx21* locus, we performed ChIP-Seq with antibodies to H3K9ac in BRD7 WT or BRD7-deficient OT-I CD8^+^ T cells from OT-I mice infected with PR8-OVA at day 8 p.i. No significant difference of H3K9ac enrichment was observed at *Tbx21* loci in BRD7-deficient cells compared with BRD7 WT cells (Figure 6A), implying that BRD7 regulated the T-bet expression independently of its binding to H3K9ac. To examine the possibility that the PBAF complex co-opts epigenetic mechanisms for target gene regulation, we further performed a ChIP assay with antibodies to H3K9me3, H3K27me3, and H3K14ac on WT and BRD7-deficient OT-I CD8^+^ T cells from OT-I mice infected with PR8-OVA at day 8 p.i. The H3K9me3, H3K27me3, and H3K14ac modifications at the *Tbx21* locus were not obviously altered after BRD7 deficiency (Figure 6E). Thus, BRD7 regulated the T-bet expression independently of H3K9me3, H3K27me3, and H3K14ac modification at the *Tbx21* locus.

### BRD7 functions as a bridge for the PBAF complex to efficiently assemble in effector CD8^+^ T cells.

**Figure 7:**
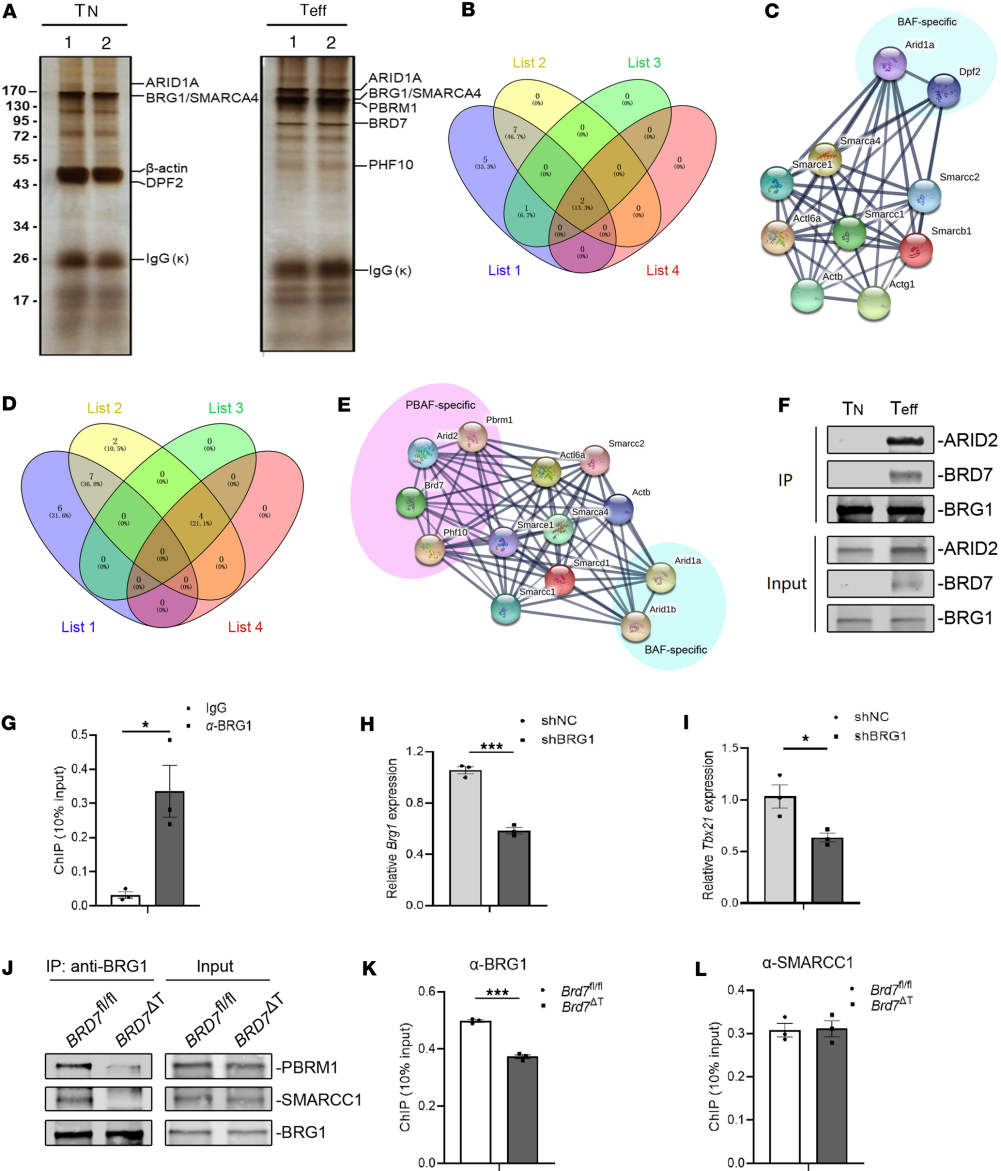
BRD7 functions as a bridge for PBAF complex to efficiently assemble in effector CD8^+^ T cells. (**A**) MS analysis of BRG1-associated proteins in naive OT-I CD8^+^ T (*n* = 2) or effector OT-I CD8^+^ T cells (*n* = 2) from OT-I mice infected with PR8-OVA at day 8 p.i. (**B**) Venn diagram showing the overlap of components. List 1: Reported components of BAF complex. List 2: Identified components of BAF complex from naive cells in **A**. List 3: Reported BAF-specific components. List 4: Identified BAF-specific from naive cells in **A**. (**C**) SWI/SNF components from naive T cells in **A** were clustered with STRING analysis. (**D**) Venn diagram showing the overlap of components. List 1: Reported components of PBAF complex. List 2: Identified components of PBAF complex from effector cells in **A**. List 3: Reported PBAF-specific components. List 4: Identified PBAF-specific from effector cells in **A**. (**E**) SWI/SNF components from effector cells in **A** were clustered with STRING analysis. (**F**) Co-IP of BRG1-associated proteins in naive CD8^+^ T (*n* = 2) or effector CD8^+^ T cells (*n* = 2) from OT-I mice infected with PR8-OVA. (**G**) ChIP (*n* = 3) shows the deposition of BRG1 at the promoter regions of *Tbx21* loci. (**H** and **I**) The mRNA expression (*n* = 3) of *Brg1* and *Tbx21* of OT-I T cells of shRNA was analyzed 8 days after transfer into recipient mice infected with PR8-OVA virus. (**J**) Co-IP of BRG1 in BRD7 WT and BRD7-deficient CD8^+^ T cells from OT-I mice infected with PR8-OVA at day 8 p.i. (**K** and **L**) ChIP (*n* = 3) shows the deposition of BRG1 and SMARCC1 at the promoter of *Tbx21* in BRD7 WT and BRD7-deficient CD8^+^ T cells from OT-I mice infected with PR8-OVA at day 8 p.i. Data are shown as mean ± SEM. *P < 0.05 and ***P < 0.001 (2-tailed Student’s *t* test). Data are representative of 2 independent experiments.

Based on the result that BRD7 regulated the chromatin accessibility of *Tbx21* locus, we assumed that BRD7 within the PBAF complex plays an important role in effector CD8^+^ T cell differentiation. To better explore the role of BAF and PBAF complexes in CD8^+^ T cell differentiation, we performed immunoprecipitation with a BRG1-specific antibody with lysates from naive or OT-I CD8^+^ T cells that were from OT-I mice infected with PR8-OVA at day 8 p.i. and were denoted as effector cells. The pull-down substrates were subsequently analyzed with mass spectrometry (MS) (Figure 7A). Being the ATPase subunit of BAF and PBAF complexes, BRG1 is shared by both BAF and PBAF complexes. Therefore, we could observe the component changes of both BAF and PBAF complexes. Interestingly, we found that BAF/PBAF-shared components appeared in the BRG1-associated proteins from both naive and effector CD8^+^ T cells (Figure 7, B–E), while PBAF-specific components, including BRD7, PBRM1, PHF10, and ARID2, existed only in those from effector cells (Figure 7, D and E). We further confirmed this result with an IP experiment and found that BRG1 interacted with BRD7 only in effector CD8^+^ T cells and not in naive CD8^+^ T cells (Figure 7F). Thus, we proposed that the SWI/SNF complexes appear merely as BAF complex in naive CD8^+^ T cells and that only the PBAF complex is assembled in the effector CD8^+^ T cells.

Because of the chromatin remodeling function, it is reasonable to assume that the ATPase unit of the PBAF complex would also be enriched at the loci of target genes. To test this hypothesis, we used BRG1-specific antibody in the ChIP assay and found that BRG1 was specifically enriched at the loci of *Tbx21* in the OT-I CD8^+^ T cells from OT-I mice infected with PR8-OVA at day 8 p.i. (Figure 7G). Based on the fact that BRG1 deficiency resulted in the loss of double-negative T cells in the thymus during T cell maturation (21), we utilized BRG1-specific shRNA (shBRG1) to specifically deplete BRG1 in mature T cells. Accordingly, the depletion of BRG1 resulted in a downregulation of *Tbx21* mRNA expression, indicating that BRG1 plays a role in regulating T-bet (Figure 7, H and I). Thus, we suggested that the PBAF complex bound to *Tbx21* loci and regulated the expression of T-bet in the effector CD8^+^ T cells.

To explore the role of BRD7 within the PBAF complex in the effector CD8^+^ T cells, we further analyzed the interactions between SMARCC1, BRG1, and PBRM1 based on the assembly process of PBAF complex in human 293T cells (17). According to this modular assembly model of the PBAF complex, the form of BAF core module and ATPase module are 2 parallel steps. ATPase module is recruited after the core module incorporation with PBAF-specific components ARID2, BRD7, and PHF10. After incorporation with the ATPase module, the PBAF complex intermediate finalizes its formation by binding with PBRM1 (17). However, this model did not propose any role played by BRD7 in assembling the PBAF complex. Since BRD7 can individually interact with SMARCC1, PBRM1, or BRG1 (17), we supposed that BRD7 may function as a bridge between PBAF core module and ATPase module. To test this hypothesis, we analyzed the interaction between SMARCC1, PBRM1, or BRG1 after BRD7 deficiency in the effector CD8^+^ T cells. We found that the binding of BRG1 to either SMARCC1 or to PBRM1 became weakened after BRD7 deficiency in the OT-I CD8^+^ T cells (Figure 7J), indicating that the binding of the core module of BAF- or PBAF-specific component PBRM1 to the ATPase module depended upon BRD7. In addition, the enrichment of BRG1 at *Tbx21* locus was significantly less after BRD7 deficiency in effector CD8^+^ T cells (Figure 7K), while the enrichment of SMARCC1 at the *Tbx21* locus was unaffected after BRD7 deficiency in effector CD8^+^ T cells (Figure 7L), implying that the recruitment of the ATPase module to the target gene depended on the assembly of the core module of PBAF with BRD7. Altogether, these results indicate that BRD7 was the bridge between the BAF core module and the ATPase module during the assembly of PBAF complex in effector CD8^+^ T cells.

## Discussion

After antigen stimulation, naive CD8^+^ T cells expand and differentiate into effector cytotoxic T cells. Multiple lines of evidence have indicated that T-bet serves as the “master regulator” of SLEC lineage commitment (5, 41–43). T-bet is highly expressed in effector CD8^+^ T cells but is lowly expressed in memory CD8^+^ T cells. It is also required for the production of IFN-γ and the cytotoxicity of CD8^+^ T cells during LCMV infection. *Tbx21* KO seriously influences the formation of KLRG1^hi^ subset of effector CD8^+^ T cells but has little effect on the IL-7R^hi^ subset (5, 42). T-bet performs these functions via influencing the expression of many lineage-specific genes in SLECs. In the current study, we have identified a prominent role of BRD7 in regulating robust development of effector CD8^+^ T cells during acute virus infection, and it functions as a key determinant of the switch from naive T cells to effector T cells. BRD7-deficient CD8^+^ T cells show impaired expression of effector molecules such as IFN-γ and Gzmb. By RNA-Seq, ATAC-Seq, qPCR, and flow cytometry assays, we found that BRD7 sustains the effector differentiation of CD8^+^ T cells directly through regulating the expression of *Tbx21*. We further demonstrated that BRD7 enriched at the *Tbx21* locus with ChIP assay. These findings indicate that BRD7 affected the development of effector CD8^+^ T cells through regulating *Tbx21* transcription.

BRD7 has been identified as a tumor suppressor gene in multiple cancers (28–30, 45–47). High expression of Brd7 is associated with improved survival in multiple cancers. Brd7 could recognize and bind to acetylated histone H3 (26). However, several studies also show that, though it did bind to acetylated histone H3, BRD7 did not have histone acetyltransferase activity (26, 27). In this study, we found that BRD7 regulated the T-bet expression independently of H3K9ac and H3K14ac modifications. We also found that BRD7 regulated the T-bet expression independently of H3K9me3 and H3K27me3 modification at the *Tbx21* locus. However, since BRD7 deficiency decreases the chromatin accessibility of *Tbx21* locus, BRD7 could regulate T-bet expression through altering chromatin modification of *Tbx21* loci as a component of the PBAF complex.

Despite many shared components, BAF and PBAF complexes are functionally distinct complexes (23). The balance between BAF and PBAF complexes was reported as a pivotal determinant of the VDR-driven antiinflammatory response (37). Recently, the researchers found that the BAF complex and c-Myc physically interact to establish the chromatin landscape in activated CD8^+^ T cells and BAF as a negative determinant of memory T cell fate (48). In our study, we describe a phenomenon that PBAF- rather than BAF-specific components only exist in effector CD8^+^ T cells, while they did not appear in naive CD8^+^ T cells. The assembly of PBAF complexes was initiated in the effector stage during CD8^+^ T cell differentiation, consistent with the significant upregulation of BRD7 expression in effector CD8^+^ T cells when compared with naive CD8^+^ T cells. The PBAF complex functions as a chromatin remodeler that includes nucleosome assembly and organization, chromatin assess, and nucleosome editing, which result in their specific interaction with particular transcription activators, repressors, and histone modifications. All these factors function together and lead to the activation of a special gene (16, 49). In the presence of BRD7, the proper assembled PBAF complex therefore enhanced the chromatin accessibility at *Tbx21* locus.

A major barrier to our understanding of the functions and tissue-specific roles of mSWI/SNF complex is the lack of knowledge of the assembly and organization of the complex. Significant progress has been made by a recent study (17). This report has posed the modular organization and assembly order of mSWI/SNF complex in human HEK293T cell line. In particular, the assembly of the PBAF complex begins with the formation of the core module including SMARCC1, and it then incorporates PBAF-specific components ARID2, BRD7, and PHF10 in order. The assembly pathway of PBAF finalizes through recruitment of ATPase module (including BRG1) and the binding of PBRM1. However, the role of BRD7 for the assembly of PBAF in this model remains to be determined. Here, we have found that BRD7 interacts with BRG1, SMARCC1, and PBRM1 and is important for BRG1 (ATPase module) and SMARCC1 (core module) interaction. Although BRD7 does not affect the loading of the core module components such as SMARCC1 onto *Tbx21* locus, BRG1 (ATPase module) binds to the *Tbx21* locus in effector CD8^+^ T cells in a BRD7-dependent manner. These results further confirmed the critical role played by BRD7 in assembling the PBAF complex in effector CD8^+^ T cells, especially at the *Tbx21* locus.

In summary, we found that the major complex of SWI/SNF chromatin-remodeling in effector CD8^+^ T cells is the PBAF complex. Despite many functions of the PBAF complex being illuminated (23), the capability of the PBAF complex to mediate SLEC differentiation is unknown. Our study shows the essential role of the PBAF complex in SLEC differentiation, and it illuminates mechanisms regarding how SLECs maintains the expression of “effector” genes in the presence of the PBAF complex. The significantly upregulated BRD7 is the key factor for PBAF assembly in SLECs. However, the signaling pathways that initiate the expression of BRD7 and the mechanism of the switch between BAF and PBAF complexes remain to be illuminated. Nevertheless, our data highlight the roles of BRD7 and its PBAF complex in the differentiation and function of CD8^+^ effector cells. By focusing on the BRD7-mediated assembly of the PBAF complex in CD8^+^ T cells, we uncovered a possible therapeutic target to interfering with the induction of functional effector CD8^+^ T cells, and this could open a new avenue for the treatment of various diseases including viral infections, tumors, or autoimmune diseases.

## Methods

### Sex as a biological variable.

Sex was not considered as a biological variable; both female and male mice were used.

### Mice.

All mice were on a C57BL/6 (B6) background. *Brd7^fl/fl^* mice crossed with *Cd4-Cre* mice (*Brd7*^ΔT^) were used. They were obtained from Shanghai Model Organisms Co. Ltd. Cre^–^ littermates were used as WT controls in all experiments. WT B6 mice (strain 000664), CD45.1 mice (strain 002014), and OT-I TCR-transgenic mice (strain 003831) were obtained from The Jackson Laboratory. OT-I TCR-transgenic mice, which use a Va_2_Vb_5_ TCR heterodimer to recognize OVAp (amino acids 257–264) presented by H-2K^b^, were congenic for CD45.1 on the B6 background. OT-I CD45.1 mice and *Brd7*^ΔT^ mice were bred, and the offspring were intercrossed to obtain OT-I BRD7–conditional KO TCR-transgenic mice. During infection experiments, WT and BRD7-deficient mice were housed together to avoid “cage bias.” No intentional method for randomization was used. Chimeras were generated by i.v. injection of 5 × 10^6^ to 10 × 10^6^ donor bone marrow cells (*Brd7^fl/fl^* and *Brd7*^ΔT^ cells at a ratio of 1:1) into lethally irradiated CD45.1 mice. *Brd7^fl/fl^* and *Brd7*^ΔT^ cells of donor origin were identified with the congenic markers CD45.1 and CD45.2. The chimeras were used at 6 weeks after engraftment. All mice were used at the age of 6–10 weeks.

### Infection with influenza A virus and LCMV-Arm.

For influenza A virus infection, mice were infected i.n. with 1 × 10^7^ plaque-forming units (PFU) of influenza virus strain H1N1, the H3N2 influenza A virus strain HKx31, or influenza virus strain A/PR/8/34–OVA (PR8-OVA). To measure recall responses, mice were first inoculated i.p. with 1 × 10^7^ PFU of the H1N1 influenza virus and then challenged i.n. with 1 × 10^4.5^ PFU of HKx31 4 weeks later. Virus stocks were grown in the allantoic cavity of 10-day-old embryonated hen eggs and stored in aliquots at –80°C. Viral titers were obtained by infection of MDCK (Mardin-Darby canine kidney) cells as previously described (50, 51). MDCK cells were obtained from American Type Culture Collection (ATCC).

For LCMV-Arm infection, mice were generally infected i.p. with LCMV Arm strain (1 × 10^6^ to 5 × 10^6^ PFU). Day 8 p.i., mice were euthanized, and donor cells were assessed using LCMV-GP^33–41^-tetramer (H-2D^b^-GP33^+^) staining.

### Flow cytometry and cell sorting.

Single-cell suspensions were prepared from spleen or lung. The following antibodies were used (all monoclonal antibodies from eBioscience): anti-CD3ε (clone 145-2C11), anti-CD4 (clone GK1.5), anti-CD8a (clone 53–6.7), anti-KLRG1 (clone 2F1), anti-CD127 (anti–IL-7Rα; A7R34), anti-CD45.1 (clone A20), anti-CD45.2 (clone 104), anti-CD62L (clone MEL-14), anti-CD44 (clone IM7), anti-CD69 (clone H1.2F3), anti-CD27 (clone LG.7F9), anti-BrdU (clone BU20A), anti–IFN-γ (clone XMG1.2), anti–TNF-α (clone MP6-XT22), anti-Gzmb (clone NGZB), anti-Perforin (dG9 [∆ G9]). Allophycocyanin-conjugated tetramers of H-2D^b^ used in the study were all from Helixgen (Guangzhou) Co.: anti-Flu.NP_366_ (H-2D^b^/ASNENMETM tetramer), anti-Flu. PA_224_ (H-2D^b^/SSLENFRAYV tetramer), and anti-LCMV-GP_33_ (H-2D^b^/KAVYNFATM).

For measurement of intracellular cytokine expression, splenocytes were isolated ex vivo and stimulated with 1 μg/mL of the major histocompatibility complex class I–restricted (MHC-I–restricted) influenza-derived peptide NP (amino acids 366–374 [ASNENMETM]) in IMDM plus 10% FBS with 50 ng/mL PMA (Sigma-Aldrich), 1 μg/mL ionomycin (Sigma-Aldrich), and 1 μg/mL brefeldin A (eBioscience) for 4–6 hours. Cells were stained for 20 minutes at room temperature with the relevant fluorochrome-conjugated monoclonal antibodies in PBS containing 0.5% BSA. For intracellular staining, cells were fixed and permeabilized with Cytofix/Cytoperm (BD Biosciences), and they were stained with antibodies against indicated cytokines. For staining of transcription factor, cells were stained with antibodies to surface antigens, fixed, and permeabilized according to the manufacturer’s instructions (Transcription Factor Staining Buffer Set; BD Biosciences). Cells were acquired on an LSRFortessa flow cytometer (BD Bioscience), and data were analyzed with the FlowJo V10.0.7 (FlowJo). The fraction of labeled cells was analyzed with a minimum 100,000 events.

For the flow cytometric sorting, a BD FACSAria III cell sorter (BD Biosciences) was used. For the isolation of H-2D^b^-NP tetramer^+^ CD8^+^ T cells from influenza virus–infected mice, single-cell suspensions of lung samples were stained with influenza virus–specific tetramers and antibodies to the relevant markers. For the isolation of naive CD8^+^ T cells from B6 mice, single-cell suspensions of spleen samples with stained with the specific fluorochrome-conjugated antibodies. All the cells were sorted with a purity ≥ 95%.

### Adoptive transfer of CD8^+^ T cells and infections.

Congenically distinct *Brd7^fl/fl^* and *Brd7*^ΔT^ OT-I CD8^+^ T cells were mixed at a 1:1 ratio and adoptively transferred at 1 × 10^5^ cells per CD45.1 recipient mouse. Mice were then infected i.n. with PR8-OVA virus.

### Generation of bone marrow chimeras.

For *Brd7^fl/fl^* plus *Brd7^fl/fl^* or *Brd7^fl/fl^* plus *Brd7*^ΔT^ chimeras, CD45.1^+^ mice were lethally irradiated with 950 rad and then injected i.v. with 1 × 10^7^ bone marrow cells harvested from *Brd7^fl/fl^* CD45.2^+^ and *Brd7^fl/fl^* CD45.1^+^CD45.2^+^ or *Brd7*^ΔT^ CD45.2^+^ and *Brd7^fl/fl^* CD45.1^+^CD45.2^+^ littermates at the ratio of 1:1. The mice were treated with sulfamethoxazole and trimethoprim (Bactrim) antibiotics diluted in drinking water for 4 weeks after reconstitution. After approximately 6 weeks, the mice were infected with HKx31 virus and sacrificed for flow cytometry analysis at 10 p.i.

### In vivo cytotoxicity assay.

Target spleen cells from B6 mice were pulsed for 30 minutes with 1 μg/mL influenza virus–derived NP peptide (amino acids 366–374) and were subsequently labeled for 20 minutes at 37°C with 0.2 μM CFSE (Invitrogen) (CFSE^lo^; specific target cells) or were not pulsed with peptide and were labeled with 2 μM CFSE (CFSE^hi^; nonspecific target cells). The 2 target populations were mixed in equal numbers, and 5 × 10^6^ cells were transferred i.n. into mice that had been infected with influenza A virus HKx31 strain 10 days before or into noninfected control mice. Mice were killed 4 hours later, and the ratio of peptide-loaded target cells to “empty” target cells was quantified by flow cytometry.

### RNA-Seq and ATAC-Seq.

CD8^+^ T cells that bound H-2D^b^-NP (NP peptide amino acids 366–374) were isolated by flow cytometry from lung of influenza virus–infected WT or BRD7-deficient mice. Total RNAs from each group were extracted by TRIzol Reagent (Thermo Fisher Scientific) according to the manufacturer’s instruction. The quality of RNA samples were evaluated by Nanodrop 2000 (Thermo Fisher Scientific) and BioAnalyzer 2100 (Agilent). The RNA-Seq library were built with TruSeq Stranded mRNA Library Prep Kit (Illumina) and sequenced with HiSeq X Ten (Illumina) at BioMarker under the PE150 protocol (52). RNA-Seq reads were trimmed, filtered, and quality controlled by FastQC (Babraham Institute) tool. The reads were aligned to mouse reference genome NCBI build 38 (GRCm38) by Hisat2, followed by calculating the reads per kilobase per million mapped reads.

ATAC-Seq was conducted with H-2D^b^-NP^+^CD8^+^ T cells of influenza virus–infected WT and BRD7-deficient mice. The ATAC-Seq library was built with TruePrep DNA Library Prep Kit V2 (Vazyme) as previously described (52). In brief, the library quality was evaluated by Qubit 3.0 Fluorometer (Thermo Fisher Scientific) and BioAnalyzer 2100 (Agilent), and data were sequenced with HiSeq X Ten (Illumina) at BioMarker under the PE150 protocol. ATAC-Seq reads were trimmed, filtered, and quality controlled by FastQC tool. Then the reads were aligned to GRCm38 by Bowtie 2, followed by rearranging with Samtools. Igvtools (Broad Institute) was used to visualize the tag peaks. Specific gene loci were amplified. Tag density from different groups was calculated by normalizing to the total mapped reads.

### ChIP-Seq and ChIP-qPCR.

ChIP was performed according to the manufacturer’s instruction (Cell Signaling Technology). In brief, naive OT-I CD8^+^ T cells or OT-I CD8^+^ cells from OT-I mice were infected with PR8-OVA at day 8 p.i. fixed with 1% formaldehyde (Sigma-Aldrich), followed by digestion with RNase cocktail. Chromatin from 5 × 10^6^ to 10 × 10^6^ cells was used for each ChIP experiment. Antibodies against normal rabbit IgG (1:50, 2729, Cell Signaling Technology), BRD7 mouse mAb (1:50, B-8, sc-376180, Santa Cruz Biotechnology Inc.), BRG1 rabbit mAb (1:50, D1Q7F, 49360, Cell Signaling Technology), H3K9ac rabbit mAb (1:50, C5B11, 9649, Cell Signaling Technology), H3K14ac rabbit mAb (1:50, D4B9, 7627, Cell Signaling Technology), H3K9me3 rabbit mAb (1:50, D4W1U, 13969, Cell Signaling Technology), and H3K27me3 rabbit mAb (1:50, C36B11, 9733, Cell Signaling Technology) were used. Antibody-DNA complexes were captured by ChIP-Grade Protein G Magnetic Beads. The immunoprecipitated DNA was purified and subjected to sequencing or PCR assessment. ChIP primers targeting the *Tbx21* were used to quantitate each target regions by qPCR.

### qPCR.

Total RNA from indicated numbers of cells were isolated with TRIzol reagent (Thermo Fisher Scientific) and proceeded to cDNA synthesis with PrimeScript RT reagent Kit (Takara). Gene expression was analyzed by Real-time PCR with SYBR Ex-taq premix (Takara) in a CFX96 Real-time PCR Detection System (Bio-Rad). Mouse β-actin mRNA was measured as internal control.

### MS.

MS analysis was performed as previously described (50). In brief, the stained bands of interest were excised into gel slices with a clean scalpel, followed by digestion with trypsin using in-gel digestion. Each gel piece was diced into small (1 mm^3^) pieces and dehydrated. The gel pieces were then incubated with trypsin (Promega) for digestion. Peptides were further extracted with 50% acetonitrile–5% formic acid, lyophilized in a SpeedVac (Thermo Fisher Scientific), and then desalted using μ-C18 ZipTip Pipette Tips (MilliporeSigma). Finally, samples were lyophilized and stored at 20°C prior to analysis by liquid chromatography–tandem MS or dissolved in 0.1% (vol/vol) formic acid–water. All samples were analyzed on a Thermo Fisher Scientific Q EXACTIVE mass spectrometer coupled with an EASY n-LC 1000 LC (Thermo Fisher Scientific) system and a nanoelectrospray source.

### co-IP and Western blot assays.

Co-IP and Western blot assays were performed as previously described (50). WT (*Brd7^fl/fl^*) or BRD7-deficient OT-I CD8^+^ T cells from OT-I mice infected with PR8-OVA at day 8 p.i. were collected and lysed. The lysates were precleared with protein A/G agarose beads (MilliporeSigma) for 30 minutes and then incubated with anti-BRG1 antibody or rabbit normal IgG antibody for 6–16 hours, followed by incubating with protein A/G agarose beads (MilliporeSigma) for 4 hours at 4°C. The beads were then washed 3 times with ice-cold lysis buffer, followed by Western blotting. The following antibodies were used: β-actin antibody (1:1,000, 4967, Cell Signaling Technology), BRD7 mouse mAb (1:500, B-8, sc-376180, Santa Cruz Biotechnology Inc.), BRG1 rabbit mAb (1:500, D1Q7F, 49360, Cell Signaling Technology), BRD2 rabbit mAb (1:500, D89B4, 5848, Cell Signaling Technology), BRD4 rabbit mAb (1:500, E2A7X, 13440, Cell Signaling Technology), TRIM28 polyclonal antibody (1:500, 15202-1-AP, Proteintech), IRDye 680RD goat anti-mouse IgG (H + L) 0.5 mg antibody (1:10,000, 926–68070, LI-COR Biosciences), and IRDye 800CW goat anti-rabbit IgG conjugated antibody (1:10,000, 926–32211, LI-COR Biosciences).

### shRNA-mediated knockdown by retroviral transduction.

DNA fragments encoding shRNA targeting mouse BRG1 (SMARCA4) were subcloned into a custom retroviral vector containing GFP as a reporter (pMKO.1). CD8^+^ T cells of OT-I mice were separated and stimulated for 18 hours in 24-well plates precoated with anti-CD3 and anti-CD28. After stimulation, cells were transduced by adding retroviral supernatants supplemented with 100 U/mL mouse IL-2 and 8 μg/mL polybrene, followed by centrifugation for 95 minutes at 950*g* at 32°C. After transduction, cells were incubated for 12–16 hours at 37°C. In total, 1 × 10^5^ transduced CD8^+^ T cells were transferred into PR8-OVA virus–infected hosts (CD45.1 mice) at day 1 p.i., and remaining cells were cultured in vitro with 50 U/mL human IL-2 for 2 days to assess for knockdown efficiency by qPCR. At day 10 p.i., CD45.2^+^ populations were assessed by flow cytometry.

### Statistics.

All data are derived from 2–3 independent experiments. Statistical analysis was performed with GraphPad Prism 5.0 software (GraphPad Software). Data are shown as mean ± SEM. Two-tailed Student’s *t* test was used to compare 2 independent groups, while 2-way ANOVA was used in multiple comparisons. Differences were considered significant when *P* < 0.05.

### Study approval.

All mice were used at the age of 6–10 weeks and were housed and maintained according to Sun Yat-sen University guidelines (permit no. SYXK [YUE] 2010-0107) (Guangzhou, China).

### Data availability.

All data sets generated or analyzed during this study have been included in this manuscript. The RNA-Seq, ATAC-Seq, and ChIP-Seq data have been deposited in the Sequence Read Archive database (accession nos. PRJNA1122236 and PRJNA1122318). The data are available in the Supporting Data Values file and from the corresponding author upon reasonable request.

## Author contributions

FH, YL, YQ, and YY designed the experiments, performed most of these experiments, analyzed the data, and wrote the manuscript; ZZ, B Luo, YW, JL, JC, and WZ performed some of the experiments; HZ provided scientific expertise and the interpretation of data for the work; and B Liu contributed to the idea generation, experimental design, and manuscript writing and conceived the project.

## Supplementary Material

Supplemental data

Supporting data values

## Version 1 07/02/2024

In-Press Preview

## Version 2 08/08/2024

Electronic publication
