# Supplementary material for: BRD7 as key factor in PBAF complex assembly and CD8+ T cell differentiation
Source: JCI Insight. 2024 Jul 2;9(15):e171605. doi: 10.1172/jci.insight.171605 (PMC11383612; doi:10.1172/jci.insight.171605)
Supplement: Supplemental data [file jciinsight-9-171605-s080.pdf]

Supplementary Material for

BRD7 Functions as a Key Factor for the Assembly of PBAF Complex and Facilitates the Differentiation of Effector $CD8^+$ T cells

Feng Huang, Yingtong Lin, Yidan Qiao, Yaochang Yuan,

Zhihan Zhong, Baohong Luo, Yating Wu, Jun Liu, Jingliang

Chen, Wanying Zhang, Hui Zhang, and Bingfeng Liu

# These authors contributed equally to this work

*Corresponding author. Email: liubf5@mail.sysu.edu.cn

This PDF file includes:
Figs. S1 to S5

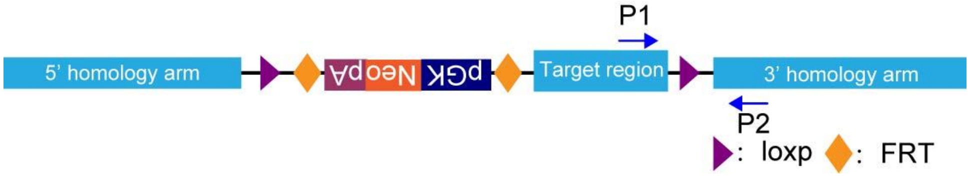

Primer	Sequence (5' →3')	Tm
P1	AAGTAGAACAGACACCCCTTCAGGAA	57.6
P2	TTGCCAGTTAAAACAGACCAAATGAG	57.6

Cycling Reaction	Step	Temp	Time	Note
	1	94 ° C	5 min	
	2	94 ° C	30 sec	
	3	58 ° C	30sec	
	4	72 ° C	1 min	34 repeats to 2
	5	72 ° C	5 min	
	6	12° C	Hold	

Result	PCR Products:Mutant:359bp ; WT:239bp. Separated by gel electrophoresis on a 1.2% agarose gel. Marker: DL2000 (Takara, Code number: D501A) 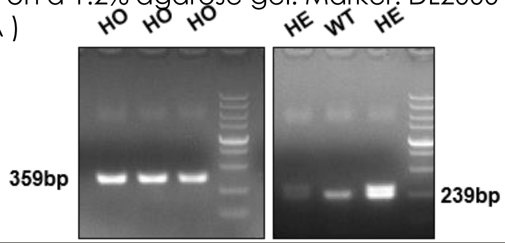
Genotype	WT: one band with 239bp; Heterozygous: two bands with 239and 359bp.

Figure S1. Construction process of the BRD7-knockout mouse.

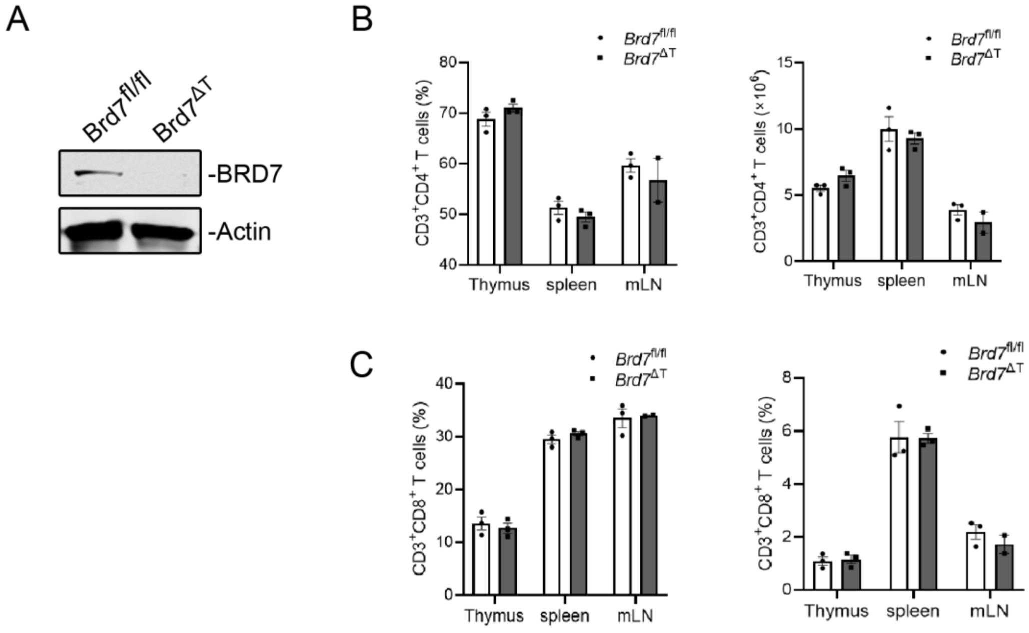

Figure S2. Removal of BRD7 in T cells with the CD4-cre deleter.

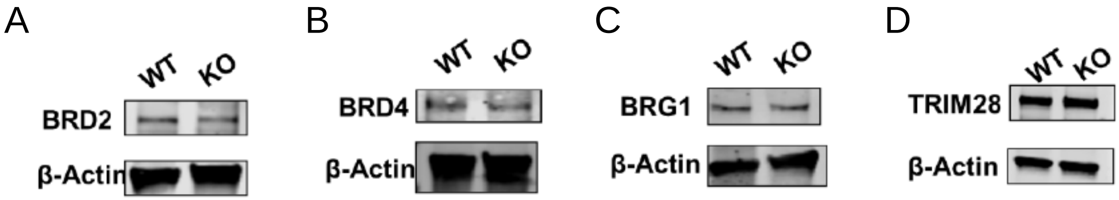

Figure S3. Wild-type (*Brd7*$^{fl/fl}$) or BRD7-deficient CD4+ T cells were collected and lysed, and subsequently subjected to western blot analysis to compare the expression differences of BRD family proteins between the two groups.

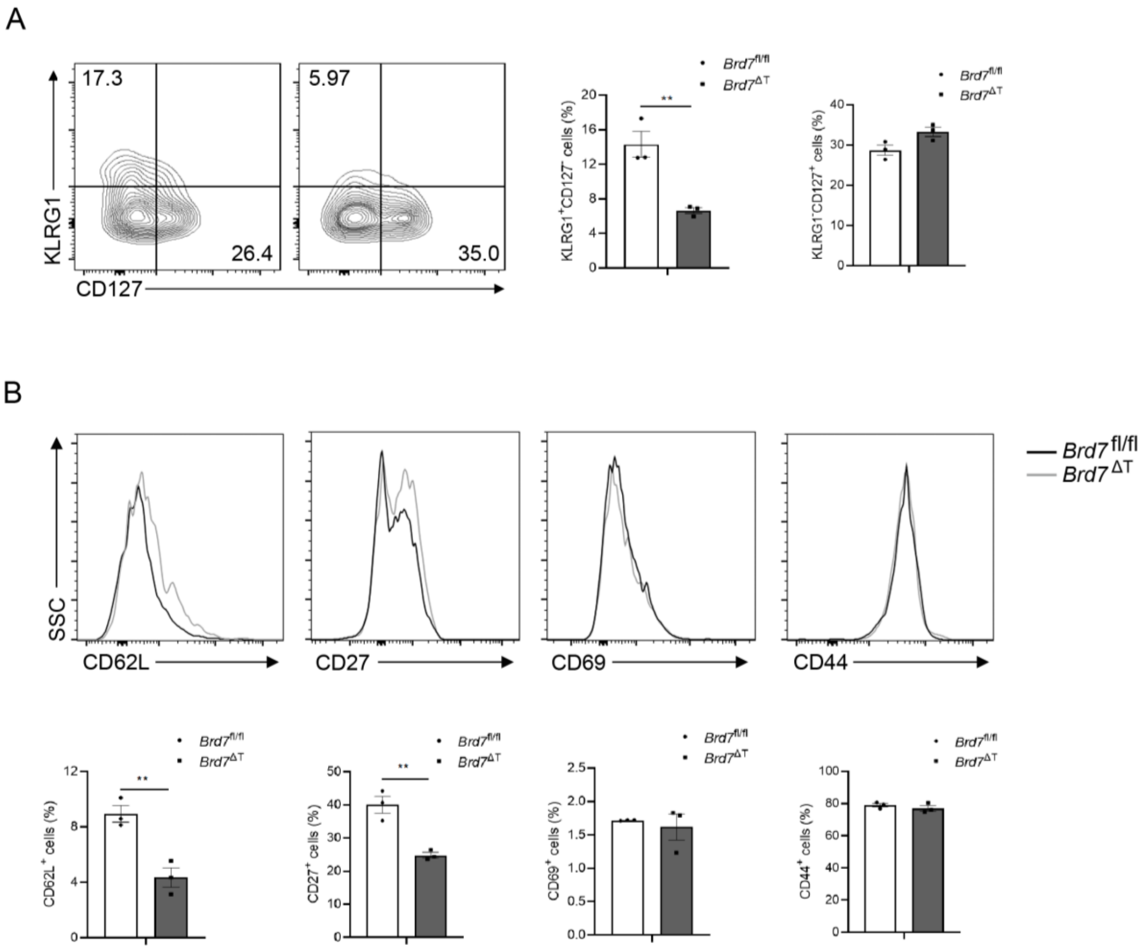

Figure S4. BRD7 deficiency impaired effector differentiation. Data are representative of three independent experiments.

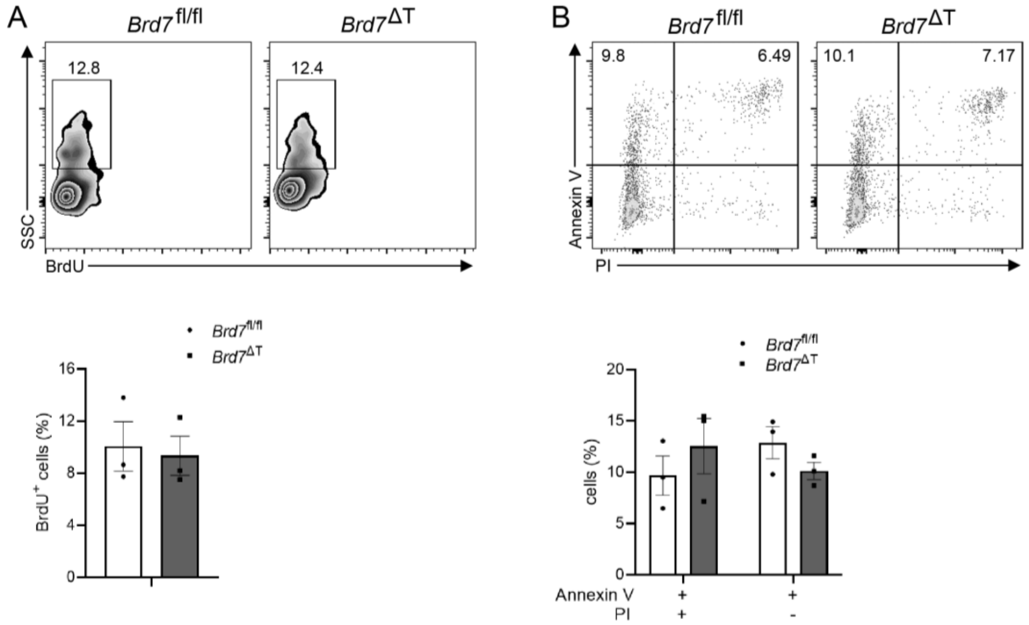

Figure S5. BRD7-deficiency did not alter the proliferation or apoptosis of antigen-specific CD8+ T cells.
